# Challenges and coping mechanisms of parents of children with attention deficit hyperactivity disorder in Addis Ababa, Ethiopia: a qualitative study

**DOI:** 10.1186/s40359-024-01828-0

**Published:** 2024-06-17

**Authors:** Wongelawit Mesfin, Kassahun Habtamu

**Affiliations:** School of Psychology, College of Education and Behavioral Studies, Addis Ababa University, Addis Ababa, P.O.BOX: 1176, Addis Ababa, Ethiopia

**Keywords:** Attention-deficit hyperactivity disorder, Children with ADHD, Parents of children with ADHD, Experiences, Challenges, Coping mechanisms, Addis Ababa, Ethiopia

## Abstract

**Background:**

Attention deficit hyperactivity disorder (ADHD) is a neurodevelopmental disorder that has manifestations of inattention, hyperactivity, and impulsivity. It affects every facet of a child’s life, including one’s own emotions, family and school life, and social interaction. The few available studies on ADHD conducted in Ethiopia focus on teachers’ awareness and the prevalence of ADHD. None of these studies has taken into account parents of children who have ADHD. The present study, therefore, aimed to find out the challenges and coping mechanisms of parents who have children with ADHD.

**Methods:**

A phenomenological qualitative study was conducted to explore the experiences of parents who have children with ADHD. The study was carried out in Addis Ababa, the capital city of Ethiopia. Fourteen parents and two healthcare providers were involved in the study. Participants were selected using a purposive sampling technique. In-depth interviews were conducted with parents of children with ADHD (*n* = 8) and healthcare providers (*n* = 2). One focus group discussion (FGD), consisting of six members, was also conducted with parents. A topic guide for conducting the interviews and FGD was developed. Interviews and the FGD were audio-recorded. The data were transcribed verbatim, translated into English, and then analyzed using a thematic analysis approach.

**Results:**

With regard to challenges of parents of children with ADHD, three themes emerged: social challenges, economic challenges and psychological challenges. Stigma is found to be the most common challenge. Other challenges included worry about the child’s future, lack of social support, strained relationships with others, impact on their job, and marital conflict. Concerning coping mechanisms, two themes emerged: Inward and outward means of coping. The inward means of coping included prayer and developing an optimistic mindset whereas the outward means were family support, healthcare providers’ guidance, and social avoidance.

**Conclusions:**

The study found that parents of children with ADHD experience several aspects of psychological, social, and economic challenges. Support from healthcare professionals, family members, and the community at large is found to be useful for parents to cope with these challenges. Future research should focus on evaluating interventions that would help parents with ADHD cope with the challenges they experience.

**Supplementary Information:**

The online version contains supplementary material available at 10.1186/s40359-024-01828-0.

## Introduction

The Diagnostic and Statistical Manual of Mental Disorders (DSM) defines attention deficit hyperactivity disorder (ADHD) as a neurodevelopmental disorder characterized by inattention, disorganization, and/or hyperactivity-impulsivity [1]. Inattention and disorganization entail inability to stay on task, seemingly not to listen, and losing materials, at levels that are inconsistent with age or developmental level [2]. Hyperactivity-impulsivity, on the other hand, refers to over-activity, fidgeting, inability to stay seated, intruding into other people’s activities, and inability to wait- symptoms that are excessive for age or developmental level [3]. These symptoms have to present prior to age 12 years, have also been present in two settings (at home and school) and they interfere with, or reduce the quality of social, academic, or occupational functioning [1]. ADHD is believed to occur in all cultures in about 5% of children and about 2.5% of adults [4]. The prevalence of ADHD worldwide is estimated to be around 6 in 100 children and adolescents and 3 in 100 adults [5, 6].

According to the World Health Organization (WHO), there are three categories of ADHD [7]. The first is the predominately inattentive type. In this category are those children with poor attention who may forget time and again, are easily distracted, sidetracked from a task, appear not to be listening, are messy, take time to initiate doing things and lose their possessions regularly. The second category is the predominately hyperactive/impulsive type. Children with hyperactivity may often be restless, fidgety, full of energy or “always on the go”, loud, continuously chattering, unable to stay seated (in the classroom, workplace, etc.), running about or climbing in inappropriate places and unable to play or do leisure activities quietly. Children with symptoms of impulsivity may often do things without thinking, have difficulty waiting for their turn in games or a queue, interrupt people in conversation, blurt out answers before the question is finished, look intrusive and start using other people’s things without permission [8]. The third one, which is the combined type, has symptoms from both the inattentive and the hyperactive/impulsive types.

ADHD has an impact on the different aspects of a child’s life, such as poor peer relationships, and low self-esteem [9]. Children with ADHD show significant academic underachievement and educational problems [10–12]. For instance, they score significantly lower on reading and arithmetic tests than controls [13].

ADHD is commonly associated with elevated levels of parenting stress because the parents’ perceptions of the demands of their role as parents exceed their resources to cope with them [14]. Stress from parenting is a set of processes that starts off from efforts to adapt to calls of parenthood and results in unwanted psychological and physiological responses [15]. Apart from higher emotional impact, impaired family activities, less parental warmth, and higher parental depression and anxiety, parents of children diagnosed with ADHD reported higher stress [16]. A study has shown that the children’s problems affect the parenting stress more than parenting stress affects the children’s problems [17]. For instance, a study showed that South African parents experienced difficulties such as negative emotions, economic problems, inadequate social support, stigma, and extra care giving responsibilities [18]. Tanzanian parents experienced difficulties in handling children whose level of functioning was impaired due to abnormal and disruptive behaviour such as not being able to follow parental instructions [19]. They are also faced with psychological problems due to caring demands exacerbated by a lack of support and stigma from the community, disruptions in family functioning and social interactions among family members.

Parents need coping mechanisms to deal with the challenges they are facing in raising a child with ADHD. Folkman & Lazarus [20] explained coping as an individual’s continuous effort in thoughts and actions to manage specific external or internal demands appraised to be challenging and overwhelming to the individual. In addition, coping is considered highly contextual, in that its effectiveness is determined by the ability to change over time and across different conditions. There is no previous study on the coping mechanisms of parents who have children with ADHD in Ethiopia. Nevertheless, a study on mothers of autistic children found that religion, experience sharing, and social support are the most commonly used coping mechanisms [21]. A similar study on parents of children with intellectual disability showed that religion (praying, fasting, and attending church ceremonies), experience sharing with like parents and maintaining smooth relations with their children’s teachers are their coping mechanisms [22].

Few studies have been conducted on the prevalence, risk and protective factors and impact of ADHD in the Ethiopian context [23–25]. Nevertheless, to the best of our knowledge, there are no studies done on the challenges and coping mechanisms of parents of children with ADHD in the Ethiopian context. This study, therefore, aimed to explore the challenges and coping mechanisms of parents of children with ADHD.

## Methods

### Study design

A phenomenological qualitative study was conducted. A qualitative approach was more suitable for this study as it seeks to acquire an in-depth understanding of the experiences of parents with ADHD through exploration instead of measurement [26]. According to Draper [27], qualitative research investigates a phenomenon considering the context of people’s everyday lives and also attempts to understand and explain the world from participants’ points of view. Green & Thorogood [28] concur by stating that the focus of qualitative research is to find explanations for questions such as “what”, “how” or “why” of an occurrence.

This study intended to assess the challenges parents of children with ADHD face and the coping mechanisms they use. Data were gathered by using in-depth and key informant interviews and focus group discussion. In doing so, the study gave participants the freedom to articulate their experiences with their own words rather than choosing words from a predetermined list. The study also adopted Bronfenbrenner’s Ecological Model as its theoretical framework [29].

### Study setting

The study was conducted at St. Paul Hospital, located in Addis Ababa, the capital city of Ethiopia. The present site of the hospital building was constructed in 1968/69 and could admit 400 inpatients and 300 outpatients. St. Paul’s Hospital opened a medical college during the Ethiopian Millennium celebration in 2007 after serving the nation as a hospital for six decades. Afterward, it was renamed St. Paul’s Hospital Millennium Medical Collage (SPHMMC) by the Ministry of Health of Ethiopia. At present, it has more than 2,500 clinical, academic and administrative staff. While the inpatient capacity is 700 beds, more than 2,000 outpatient and emergency clients visit the health facility every day. St. Paul Hospital has a vision of becoming a medical university with a prestigious academic and research center, and one of the most sought- after medical care providers [30].

The Child and Adolescent Psychiatry Department of the hospital launched its service around 8 years ago. It provides services to children and adolescents who have different types of psychiatric conditions such as depression, autism spectrum disorder, ADHD, intellectual disability, substance use disorder, oppositional defiant disorder, and conduct disorder. On average, around ten patients pay a visit to the department daily. Service users will have their follow-ups until age 18 years in the Department and then will get transferred to the Adult Psychiatry Department. There are two resident psychiatrists, one senior psychiatrist, two psychologists and two nurses who are providing services in the Department.

### Participants

Purposive sampling was used to select participants in the study. This was carried out until saturation was reached. Parents who have children with diagnosed ADHD and healthcare providers who are providing treatment to children with ADHD participated in the study. Participants had to meet the following inclusion criteria to be included in the study: being a parent to a child with a diagnosis of ADHD, ability to communicate fluently in Amharic, and willingness to participate in the study. As for the healthcare providers, the study included the two of them who were on duty during the study. The target population of this study was parents of children who have ADHD at St. Paul Hospital and health care professionals who were providing services to these children and their parents. The nurse provided the information on whether parents had children with ADHD or not. Then, parents were asked for their oral consent to participate in the study.

There is no formula to acquire a sample size in qualitative research. Rather, most scholars agree on the concept of data saturation to reach to sample size. Englander [31] argued that sample size in qualitative research is often determined on the basis of theoretical saturation (the point in data collection when new data no longer bring additional insights to the research questions). Sandelowski [32] suggested that the assessment of the sample size’s appropriateness becomes a “matter of judgment”, depending on the milestones retained in the attention field by the researcher.

Creswell [33] recommended interviews with up to 10 people in phenomenological research so this study planned to interview two healthcare professionals and eight parents who have a child with ADHD. Regarding the focus group discussion, Johnson & Christensen [34] suggested that focus groups usually contain 6–12 persons. Krueger [35] suggested 6–9 focus group members and groups with more than 12 participants tend to limit each person’s opportunity to share insights and observations while focus groups with less than 6 participants make it difficult to sustain a discussion. One focus group discussion was conducted comprising six parents who have a child with ADHD.

### Methods and procedures of data collection

In-depth and key informant interviews and focus group discussion (FGD) were used as methods of data collection. Both interviews and FGD gave the participants the autonomy to express their experiences in raising children with ADHD. In-depth interviews and FGD were conducted with parents of children with ADHD. Healthcare professionals who diagnose and provide treatment to children with ADHD at St. Paul Hospital were also interviewed. Triangulation is of vital significance in qualitative research in terms of data collection method and data source. Having different respondents for the interview and FGD enriched the information gathered. The sequence for the data collection was from individual interviews with parents to focus group discussion with parents and then to individual interviews with healthcare providers. The major reason for this sequence was that in-depth interview with parents was the primary method of data collection and it was easier to make the interview with parents iterative than the interview with healthcare providers as well as the FGD with parents. In addition, the interview with parents shaped both the FGD and the interview with healthcare providers.

We developed a topic guide for the interviews and the focus group discussion (Supplementary Material 1). The questions within the topic guide we used for parents focused on finding out parents’ reactions to the first diagnosis of their child, the change after diagnosis, their positive experiences, the challenges they face and their coping mechanisms. For the healthcare providers, the questions focused on finding out about parents’ reactions to the first diagnosis of their children, the challenges of parents and the support given by the healthcare providers. For each guiding question, planned probes were included.

Interviews involve a one-to-one in-depth discussion where the researcher adopts the role of an “investigator.” This implies the researcher asks questions, controls the dynamics of the discussion, or engages in dialogue with a specific individual at a time [36]. According to Nyumba [36] in a focus group discussion, researchers adopt the role of a “facilitator” or a “moderator.” In this setting, the researcher facilitates or moderates a group discussion between participants and not between the researcher and the participants. The study was conducted in a naturalistic setting of the out-patient of the Department of Child and Adolescent Psychiatry at St. Paul’s Hospital. Both the interviews and focus group discussion were conducted in Amharic and electronically recorded. This allowed the researcher to refer to the data gathered anytime and also avoid recall bias. In-depth interviews with parents lasted between 30 and 50 min, whereas the FGD took 90 min. The key informant interview with one of the healthcare providers lasted 45 min and with the other 60 min.

### Data analysis

Data were transcribed verbatim and then translated into English. We followed the following four steps in analyzing the data: (a) familiarization with the data, (b) generating initial codes and searching for themes, (c) reviewing themes, and (d) naming themes [28].

In the course of getting familiar with the data, we listened to the audio recordings several times and transcribed the recordings into text format. Then meticulous reading of the transcripts was carried out with the aim of spotting keywords or phrases describing the experiences of the informants. Labeling and organizing the relevant pieces enabled the coding to be achieved which subsequently helped in identifying key themes. The formation and naming of these key themes were a result of the grouping of related themes. Then the subthemes were formed.

### Ethical considerations

We confirm that the study was conducted in accordance with the Declaration of Helsinki. Ethical approval for the study was obtained from the Research Ethics Committee of the School of Psychology, Addis Ababa University, and the Ethics Committee of St. Paul’s Hospital Millennium Medical College. The study was carried out in a manner that was transparent to all the participants. All the participants in the study were well informed of the aim of the study. Only those who gave their oral informed consent to participate in the study were included. The participants were assured that the data gathered would only be used for research purpose. In addition, they were reassured that the use of the voice recording was solely for the research.

Assurance of confidentiality was attained by giving pseudonyms to participants. At the end of the interview, all participants were debriefed. Finally, the researcher offered the chance of getting the findings of the research to the participants.

## Results

The socio-demographic characteristics of the interview and FGD participants are presented in Table 1.

**Table 1:** Socio-demographic characteristics of participants

Socio-demographic characteristics of the interview participants					
Adopting or biological parent	Pseudonyms	Marital status	Religion	Educational level	Occupation
Biological mother	Tayitu	Married	Orthodox	7th grade	Stay-at-home mom
Biological mother	Menen	Married	Orthodox	11th grade	Stay-at-home mom
Biological mother	Deraretu	Married	Protestant	10th grade	Cleaner
Biological mother	Mentiwab	Married	Protestant	11th grade	Stay-at-home mom
Biological father	Haile	Married	Protestant	BA degree	Pastor
Biological mother	Tirunesh	Divorced	Protestant	Cannot read and write	Tea and coffee vendor
Biological mother	Kedija	Married	Muslim	10th grade	Stay-at-home mom
Biological mother	Fatuma	Married	Muslim	10 + 1	Stay-at-home mom
**Socio-demographic characteristics of the FGD participants**					
Biological mother	Muluemebet	Married	Orthodox	12th grade	Self-employed
Biological father	Workneh	Cohabiting	Orthodox	Diploma	Surveyor
Biological mother	Mesert	Married	Protestant	10 + 2	Stay-at-home mom
Biological mother	Berhane	Single	Orthodox	BA Degree	Teacher
Biological mother	Sifan	Married	Muslim	12th grade	Stay-at-home mom
Biological father	Afework	Married	Orthodox	BA Degree	Teacher

The in-depth interview participants were eight in number and all were biological parents. Seven mothers and a father participated in the interview. All were married, except one (who was divorced), in terms of marital status. The age of the parents who participated in the interview ranged from 27 to 48 years. Concerning their religion, two of them were Muslims, two of them were Orthodox Christians and the rest four were Protestants. With regards to educational level, one was not able to read and write, two were seventh graders, three were tenth graders, one was eleventh grader and one had a BA degree. When it comes to occupation, five of them were stay at home mothers, one a cleaner, one tea and coffee vendor and one a pastor. Respondents of the focus group discussion were six biological parents. The group consisted of four mothers and two fathers, with age ranging from thirty to sixty. When it comes to marital status, there was one single parent, four married and one cohabiting with a partner. Four of them were orthodox Christians whereas the other two were Protestant or Muslim. Regarding their educational level, two of them had first degree, one had diploma, two of them completed 12th grade and one was a tenth grader. Concerning their occupation, the two degree holders both were teachers, one self-employed and three of them were stay-at home mothers.

Concerning the socio-demographic characteristics of health care professionals, one was a female General Practitioner and the other was a male third-year Psychiatry resident. Both were single. In terms of religion, one was Muslim and the other Protestant.

The major themes along with their sub-themes that emerged from the data are presented in Table 2.

**Table 2:** Major themes and sub-themes

Major theme	Sub-themes
Reaction to child’s first diagnosis	• Acceptance • Shock and confusion • Emotional ambivalence
Parental change after diagnosis	• Awareness about ADHD • Care • Change in oneself
Positive experience of parents	• Knowledge • Inspiration • Advocacy
Challenges of parents	• Social challenges • Economic challenges • Psychological challenges
Coping mechanisms	• Inward means • Outward means

### Parents’ reaction to their children’s diagnosis

For the question about their reaction when the healthcare provider first informed them about their children’s diagnosis, parents responded that they reacted with a range of feelings. There was no ‘right’ way to feel to come to terms with the diagnosis and move on with everyday life. The healthy thing to do was to recognize and validate these feelings. Some accepted it, some were shocked and confused and others were engulfed with different feelings. Apart from those who accepted it easily, we had observed other parents responding with sadness and teary eyes or even sobbing.

#### Acceptance

Parents’ acceptance of their children’s diagnosis is a way of melding the condition in their lives without engaging in any intrapersonal conflict. Intrapersonal conflict was a fight within oneself with one’s thoughts and values. The intrapersonal conflict for parents like these might be in the form of self-blame, guilt, blaming others, becoming mad at God, or taking the incident as a punishment from God. Easy acceptance of their children’s diagnosis was possible for some parents because of the prior information they got from different sources.

*While we were living in Saudi Arabia, I had seen TV programs that enabled me to have a clue concerning children’s mental health. So accepting it was not that much of a trouble to me. (INTV, Fatuma)*

*As a teacher, I knew the behaviour of children. I had noticed something wasn’t right when it came to the behaviour of my son. I couldn’t understand why he didn’t have the motivation and the patience to study. I searched over the internet and finally took him for an assessment. (FGD, Afework)*

#### Shock and confusion

Hearing the unexpected news yielded two predominant emotions: shock and confusion. Shock was a reaction to a surprising and unpleasant incident while confusion was a state where one feels disoriented, cannot think clearly and is unable to make a decision. Two of the interviewed mothers had the following to say:

*My daughter was on Thyroxine tablets that were prescribed to her by doctors in Saudi Arabia. Back then I was told that my daughter’s behaviour was a result of the side effect of this medication. But when we came to Ethiopia, she was diagnosed with ADHD. As I had accepted what the doctors from Saudi Arabia said about the behaviour of my daughter, finding out she had a mental health condition was shocking. The two different opinions of the doctors made me confused. Why the doctors did come up with two different diagnoses? (INTV, Sifan)*

*I didn’t expect my daughter would be diagnosed with a mental health condition so it was shocking news for me. I raised my daughter alone and struggled to support us. God knew what I was going through and had helped my problems come to pass. So, when this happened I was confused why God allowed another problem to happen to me. I had seen no hope until my daughter started taking the medication. (INTV, Tirunesh)*

#### Emotional ambivalence

Some of the parents reported that they experienced emotional ambivalence (a state where one has a mixture of emotions that might contradict each other). These included anxiety, hopelessness, depression, guilt, self-blame, denial, shame, self-doubt, sadness and blame. Anxiety arises from the feeling of uncertainty and fear about what the future holds for their children. Blaming others, guilt and self-blame were effects of the search for possible causes of the disorder. Parents moved down memory lane to dig out what they should have done. Anxiety, fear and insecurity could lead to denial about the incident. Feelings of shame arise when parents think about what society might say about their child’s diagnosis. Parents also might doubt themselves about their competence as a parent.

The news about the diagnosis erupted mixed emotions in other parents which included denial, anxiety, sadness, hopelessness, shame and self-blame. One mother whose son had epilepsy explained the assurance they had in prayer for the spiritual healing of their son. Not seeing any progress made her sought professional help. He was not only diagnosed with epilepsy but also with ADHD. What she had felt was anxiety, hopelessness, confusion, shame and self-blame. This was what another sobbing mother had to say:

*My son had swallowed a coin and we took him to the hospital. X-ray images were taken thrice. I blame myself for not refusing when the medical practitioners did that. So I believe the exposure to the X-ray that day caused my son’s ADHD. I felt as though I’d failed so I blamed myself for that*. *In addition, I had shame, denial and guilt. (FGD, Mesert)*

A father had also the following to share:

*As our neighbors told us, my son had fallen several times when he was very little and we believe that his ADHD was a result of it. A teacher was complaining about my son’s behaviour to his mother. His mother told the teacher that he had started holy water treatment for his behaviour. The principal of the school overheard their conversation and later on warned us that he must get assessed by a professional. As the school is an international school, she also wanted a medical certificate after his assessment in order for them to support him afterwards. I had felt shame and sadness. (FGD, Workneh)*

The two healthcare professionals described what the reactions of parents were when they were first told about the diagnosis of their children. The resident psychiatrist reported that most parents got confused, shocked, and exhibited denial.

The general practitioner shared her observation as:

*Most of the parents did not have a clue about ADHD and often were perplexed and assumed ADHD was as a full-blown psychosis. Moreover, parents wrestled with self-blame if something had happened in the past which they might think had caused their child’s disorder. This included a fall of the child that took place during the early years, instances during their pregnancy, etc. (INTV, GP)*

### Parental change after diagnosis

Parents reported that they experienced a change in themselves after they knew about the diagnosis of their children. These included an increased understanding of ADHD and better care provision.

#### Awareness about ADHD

For some of the parents, the change after the diagnosis of the child was an increased understanding about the condition not only for themselves but also for the ones that were close to them. Two parents shared the changes that took place with them as follows:

*I thought my son’s condition was associated with supernatural evil manifestation and that was the reason why we chose prayer prior to getting him professionally evaluated. Our perspective has shifted after his diagnosis. (INTV, Deraretu)*

*The first time I heard about ADHD was in the psychiatrist’s office during my son’s assessment. When I go home, I shared the information with my wife. Later on, I told my elder sister about it as she sometimes looked after him. (INTV, Haile)*

#### Care

Knowing about their children’s condition has enabled some parents to become better caregiver to their children. One parent described it as:

*I not only give him unique attention to his needs but also strive for his emotional well-being. I am always more watchful when relatives come to visit us so that they don’t call him “naughty” or something else. Whenever I had to go pay a visit to a relative, I wanted to take my other kids along and leave my son home. On the one hand, I feel like I am protecting him from harsh comments. On the other hand I knew this would make him feel lonely. In the end, I refrained from doing this and went by myself. (INTV, Tayitu)*

Another parent added:

*I came to know that physical exercise helps with concentration. I always encourage my son to do regular physical exercises. (FGD, Afework)*

### Positive experiences while parenting a child with ADHD

Even though their situation was unfortunate and dubious, parents gained some sort of positive experience in the upbringing of their children with ADHD. These included knowledge, advocacy, and inspiration.

#### Knowledge

For most of the parents, their knowledge of ADHD increased after the diagnosis. Moreover, they shared what they knew with the ones they believed should know about their children’s condition as described in the quotations below.

*A teacher had called my son stupid which had been informed to the director. As the teachers didn’t have a clue about ADHD, I made a brief explanation about his condition to all of them. (INT, Haile)*

*Fellow teachers used to tell me to support my son academically as if I was negligent towards this. I tutored a lot of children and enabled them to be high achievers academically. I knew my son had a good potential but the perspective of others differed about it. I shared what I knew about the condition with a heavy heart knowing it was not a one-time incident. I wish people were aware of ADHD. (FGD, Afework)*

#### Advocacy

Becoming an advocate for children with ADHD was another positive experience gained for most of the parents. Parents have become advocates with the intention creating a supportive environment for their children. The quotation below supports this narration.

*If I see children who have similar behaviour as my son’s, the first thing that comes to mind is his condition (ADHD). Whenever I take a taxi and observe a hyperactive child, I try to play with them and defend them if any negative comment is forwarded. (INTV, Kedija)*

Another parent claimed that he not only became an advocate for children with ADHD but also for mental health in general.

*During a discussion about mental health, I made sure people dropped any myths they might have about ADHD and other mental health issues. Even the ones we considered worst like schizophrenia can be managed with medication and normal life can be attained. (FGD, Workneh)*

#### Inspiration

Some of the parents wanted to share their experiences with other similar parents and also get encouraged by other parents who have children with other neurodevelopmental disorders. A parent highlighted her positive experience in terms of knowledge of ADHD, inspiration, and advocacy as follows.

*While waiting for my son’s turn at the hospital, I always engage in a conversation with other parents. If the parents have a newly diagnosed child, I tell them their journey might be challenging but it’s manageable. I told them how I managed my son’s epilepsy and ADHD. If their child has autism or another condition, it’s my turn to learn from their strength. (FGD, Mesert)*

### Challenges of parents of children with ADHD

The study found that parents of children with ADHD experienced psychological, social, and economic challenges. More specifically, these included worry about the future of the child, stigma, lack of social support, strained relationship with others, impact on their job, marital conflict, teachers’ lack of knowledge on ADHD, and having anxiety and depression symptoms.

#### Social challenges

Social challenges were those challenges originating from society and have negative consequences on the individual. Stigma was found to be one of the major social challenges for the majority of the parents. This included a negative, biased and unfair belief about ADHD. An excuse for laziness, way out of trouble, wrongly disciplined, and judgment on parents were some of the aspects of stigmata associated with ADHD.

Less social support from relatives and neighbors and a low level of knowledge of teachers take up the next level of challenges. The less social support from relatives and neighbors might be the result of stigma. Strained relationships and marital conflict are the challenges that stood out for a few parents in addition to other social challenges. Due to the stigma and social avoidance, some parents had strained relationships with members of their community. Others had conflicts with their partners regarding the diagnosis and treatment of their child’s ADHD.

A parent stated her experience as:

*I face different setbacks but the one that hurts me most is my marital conflict. My husband doesn’t seem to care about my son who has ADHD. Ever since my son’s diagnosis, he isolated himself from anything connected to my son’s condition. In regards to my son’s school, the school administration informed me that my son wasn’t up to the criteria set for kids with autism and therefore had to leave the school. I took the case to the Ministry of Education. So I carry the entire burden by myself. (FGD, Muluemebet)*

Another mother added:

*I have a similar story. My mother-in-law believes that my son’s condition is brought by evil spirits and the remedy is in the hands of a shaman. As she has inflicted this idea in my husband’s head, a fight arises whenever my son’s condition is raised. One day my son was late to come home. But when he returned, his father locked the door and started beating him up. He opened the door to let him out on the arrival of the police. Even though I had explained to my sisters about my son’s condition, they suggested I should look for a detention facility for children like him. Due to my son’s condition, I no longer work and am a housewife. I sometimes get judged about my son by individuals from my church’s congregation. (FGD, Mesert)*

#### Economic challenges

Economic challenges were those challenges that put economic turmoil on an individual due to mental health conditions. Quitting job to take care of their children with ADHD was the challenge to many of the parents. As taking care of children with ADHD demands a lot of patience, parents especially mothers fear other caregivers might lose their temper and hurt their children, and, therefore, quit their job to look after these children. Few parents reported the stress they had because of their socioeconomic status and others terminated their children’s follow-up due to financial strain. Overall, parents of children with ADHD described the impact on jobs and expenses associated with the child’s illness.

Spending extra time on school-related activities, such as helping the child to do homework and assignments was found to be difficult and frustrating not only for the child but also for the parent as well. A parent stated that her life is always nothing but stress. This parent carries the burden of being a single mother, divorced, financially constrained, and a daughter with a mental health condition who always worries about the actions of her landlord and stigma.

*My husband abandoned me the minute our daughter had her first seizure. She no longer has seizures but I have never heard from him. Because of her behaviour, I got to relocate and met new landlords frequently. As I am the sole breadwinner, the financial constraint is enormous. With the earnings I get from selling tea and coffee, meeting my needs of living expenses coupled with my daughter’s follow-up is so nerve-racking. Moreover not knowing her condition, people always judge me for not raising a well-behaved child. (INTV, Tirunesh)*

### Psychological challenges

Psychological challenges as a result of being a parent of children with ADHD which included stress, anxiety, sadness, loneliness, helplessness, hopelessness, and depression were frequently aired in the interviews and FGD. Most of the parents worried about their child’s future. Parents worried a lot about who would have the patience to take care of these children when they were no longer alive. A mother claimed she had been depressed for some time until recently. This mother felt helplessness and had no happiness in life.

Here is what two mothers had to say about the psychological challenges of parents of children with ADHD.

*I was in a queue waiting for a taxi with my daughter. She wanted to play around but I refused to let her go because I feared a car might hit her. She knelt, started crying and would not stand up. One guy interfered and asked me why she was crying. He was so suspicious that I abducted someone else’s child. An ugly scene was created which later involved the police. I explained her condition to them and the case was solved. I was humiliated. Another issue that I face constantly is with my daughter’s handwriting. Her teachers said that her handwriting is below her peers. Her teachers not only complain about her behaviour but also her handwriting. This deeply made me sad and angry. (FGD, Sifan)*

*I constantly worry a lot about my son. I always beg my son not to go out of our compound but boredom seems the rationale for him to do so. Some boys have got sexually molested near where we live. My prior warnings and advice I gave him didn’t seem to work as I had to tell him time and time again. (FGD, Mesert)*

The first author witnessed one of the parents who participated in the interview throwing the appointment card on the nurse’s desk and shouting at her which later ended up in crying. The interviewer calmed her down and she said she was so stressed out. According to her, nurses in the other department mistreated her and people were judging her. She later apologized to the nurse for the way she behaved.

When it comes to the challenges that parents of children with ADHD faced, the resident explained that they faced stigma, hopelessness, and depression.

The general practitioner added:

*ADHD affects every aspect of the lives of these parents. The significant challenge that parents face is at the school where these children go to. Unless they have comorbid conditions, children with ADHD go to regular schools and are bombarded by the harsh criticisms and judgments of their teachers who lack knowledge about ADHD. In addition, managing the child’s behaviour daily, stress, concern about the future of the child, impact on their job and marital conflict were other challenges that parents faced. Upon discovering heredity as being one possible etiology of ADHD, most couples argued over who passed it on to the child and mothers ended up taking the blame mostly. Some couples don’t reach an agreement on whether to continue with the follow-up or not. This mostly resulted in the discontinuation of the treatment. Some fathers also expressed feelings of doubt about the mother’s competence as a parent. Sometimes the diagnosis of the child involves not only the parents but also in-laws. (INTV, GP)*

Some parents whose children had become teens explained another psychological challenge as:

*My son used to take his medication properly. The doctors had increased the dosage of his medication. I was the one who gave him the medication every night. By the time he became a teen, he started refusing to take his medication and going for follow-ups. My wife and I couldn’t force him to continue and we are waiting on him to start again. The journey takes a different lain when children with ADHD become adolescents. (FGD, Afework)*

*My son doesn’t want to go to the hospital now. The different mental conditions of the children he saw at the hospital tormented him emotionally. By the time he became a teen, he claimed he wasn’t as mentally ill as the others and did not see the point of going to the hospital. As the medication had made a huge difference for him, I did not want him to stop taking it. So I usually go to the hospital without him for the prescription. I usually inform the doctors about his status and any change in his behaviour if any and they adjust the dosage accordingly. I wanted to have more kids but changed my mind after his diagnosis. (INTV, Menen)*

### Coping mechanisms of parents of children with ADHD

Parents reported different types of coping mechanisms they use to deal with the challenges they face when raising children with ADHD. Parents used a combination of different coping mechanisms which could be broadly grouped into two: inward means and outward means.

#### Inward means

The inward means were the mechanisms that the parents perform to come up with a positive outlook. These included prayer and the state of being an optimist. Prayer decreased stress, gave a sense of relief, improved self-esteem, and increased spirituality. Due to challenges like isolation and stigma, prayer was a way to connect to their Creator where parents feel their worries and fears are heard without judgment and validation for what they go through. Being optimist during difficult times helped with handling stress and boosted resilience.

Prayer was practiced as the first coping mechanism by almost all parents. Being optimistic was another coping mechanism for other parents. Below are what three parents had to say about their use of inward coping mechanisms:

*I observed my daughter grasping something faster than her siblings and knew she had potential. So I believe that Allah has created her for a bigger purpose in life. I pray and lean on Him to help her be what He wants her to be. (FGD, Sifan)*

*With a lot of challenges that are going on in my life, prayer is the one thing that calms me down. As my elder sisters are living within the same compound, we have daily prayer. I pray about my son a lot. I believe God has a bigger purpose in my son’s life. (FGD, Mesert)*

*I don’t know what I would do without prayer. Being a single mom with a mentally ill child, facing financial constrain, and living in a hypercritical society is so exhausting. When I pray to God for my provision, He miraculously makes it available. (INTV, Tirunesh)*

*Even though I am faced with different challenges, I am trying to see the bright side of life. Whenever I take my child for a checkup, I get a chance to observe other mothers who have children with autism or other disorders. My challenges mean little when compared with theirs. This makes me grateful and hopeful. (FGD, Sifan)*

#### Outward means

The outward means referred to coping mechanisms that parents get from their environment. These coping mechanisms included family support, healthcare providers’ guidance, and social avoidance. Every appointment gave a chance to the parents to get little information. Few but very close individuals knew about their child’s condition and give support to these parents. Others use avoiding social gatherings and interactions as a coping mechanism. In fear of the stigma, these parents had decided to meet up with only those who knew their child’s condition. Some even did not have friends that they can talk to.

Some parents reported that guidance from healthcare providers was one of the most useful coping mechanisms. Healthcare providers’ guidance in combination with other coping mechanisms was used by many parents. Some parents also used family support as a coping mechanism.

*I haven’t told anyone about my son’s condition except my sister who is supportive of me. I don’t allow my son to go out and play with kids from the neighborhood for fear of being called names and getting bullied. I had observed a few kids who exhibited similar behaviour as my son and I believed their parents kept their conditions undisclosed. I therefore did the same thing and kept it a secret. My son had a seizure in the beginning and people had compassion for such illness not for his ADHD. Due to this, I prefer my son’s seizure over his ADHD. I wish people would stop being judgmental towards any illness. (INTV, Tayitu)*

A mother expressed how the assistance she got from the health care providers became one of her coping mechanisms as

*In the beginning, my husband and I were so much focused on prayer as a solution to our child’s problem. But after a while, the continuous aid from the healthcare providers became one of our coping means. (INTV, Deraretu)*

A mother used social avoidance as a coping mechanism and expressed it as

*I take him [the child with ADHD] anywhere I go like grocery shopping and other places he shouldn’t supposed to go like funerals. I have avoided meeting up with my friends. My parents’ place is a safe haven for my son and me. I am longing to see a support group with whom I can talk freely without being judged. (INTV, Fatuma)*

## Discussion

The study shows that the reaction of parents during and after the diagnosis of their children varies from simple acceptance to a mixture of different emotions. Some of the parents noticed deviant behaviour in their children and got their children assessed due to their doubt and later on accepted their child’s diagnosis easily. Due to prior knowledge they have had on ADHD, these parents accepted the diagnosis easily as it gave them relief regarding their doubt. This finding is similar to other studies done on ASD diagnosis in a way that parental suspicion of a child’s developmental problems leads to getting an early diagnosis and coping more with the diagnosis than those not suspicious [37, 38]. A study by Dosreis et al. [39] had a similar finding on parents of children with ADHD that acceptance was achieved by 38% of parents as their main concern was trying to find an explanation for the difficult behaviour of their children.

For parents whose children have been diagnosed with mental illness, resolution is a fundamental part of the process en route to acceptance [40, 41]. According to Pianta and Marvin [42], resolution can be taken as accepting the diagnosis and integrating it into one’s life while refusing to accept self-blame. Milshtein et al. [40] argued resolution is a perception of complying and acknowledging the diagnosis and its inference. For parents of children with ASD, acceptance and normalization are very important to give the best possible life to their children [21].

Upon finding out about their child’s diagnosis, the majority of participants flaunted their reactions with a variety of emotions which include hopelessness, confusion, shame, guilt, self-blame, confusion, anxiety and denial. This is consistent with the findings of other previous studies done on the commonly experienced emotions about a child’s mental health diagnosis which include helplessness, devastation, sadness, loneliness, guilt, anxiety, and grief [43, 44]. Parents of children with mental illness go through a feeling of loneliness, misunderstanding, stigma and rejection, grief and self-blame, cynicism, unhappiness, guilt, and anxiety [44]. In congruence with other studies [40, 41], the findings of this study showed no relationship between the reactions of parents to diagnosis and parental demography. Fathers and mothers of children with ADHD reacted similarly.

The positive experiences parents gain while raising children with ADHD include knowledge, advocacy, and inspiration. Consistent with our study, Ustilaite and Cvetkova [45] revealed that parents of children with disabilities gained a range of positive experiences like inner parental growth, family relationships, finding new spiritual and material resources and feelings such as love, emotional bond with the child, and child as a source of joy and happiness.

Parents who are involved in this study experience different types of psychological, social, and economic challenges while raising children with ADHD. From these, parents’ concern for the future well-being of children and stigma from the community stood out. Social challenges include stigma, limited social interaction, marital conflict, strained relationships, teachers’ lack of knowledge of ADHD, and low social support. According to Sirey and colleagues [46], stigma is a socially formulated observable fact that encompasses stereotyping, labeling, segregation, loss of status, and nepotism which are allowed to take place in social circumstances by individuals with power. A “culture of suspicion”, about mental health treatment particularly if it involves a child, has been created by the stigmatizing convictions towards people with mental health conditions [47].

The finding that some parents have challenges in their social interaction and have strained relationships is similar to a previous study [48] which found that children’s ADHD has negatively influenced parents’ social lives and forced them to have frictions in their relationships. Other similar studies have revealed that parents had feelings of isolation from their friends and families, due to other adults being intolerant of their children’s behaviour [18, 49]. In regards to marital conflict, this study has similar findings to other studies [18, 50] which found that it is a result of unlike opinions among parents on the diagnosis and treatment of their child. Other studies revealed that it might be caused by troubles with a child’s behavior [51]. In a study conducted by Wymbs et al. [52] parents who had a child with ADHD were not only more likely to divorce but also had a shorter latency to divorce than parents of children without ADHD.

Ambikile and Outwater [53] found that the challenges of Tanzanian parents who have children with mental disorders including ADHD were insufficient children’s social services, stigma, childcare strain, lack of public awareness of mental illness, absence of social support, and troubles with social life. As per the different studies conducted in Ethiopia [54, 55], a large number of teachers lack knowledge on ADHD. This negatively impacts the parents and that is what the present study identified as one of the challenges for the parents.

A study carried out in Ethiopia on parents of children with ASD revealed that parents have a social burden [56]. Other similar studies [57, 58] found that the challenges of parents of children with ASD were marital conflict, time-consuming, lack of social support, stigma, the severity of the child’s behaviour, child’s inability to understand feelings and needs, inadequate service (school and treatment), and lack of self-care. In another study, separation from a partner, family/societal reactions, and social isolation are the challenges of parents who have children with intellectual disability [59].

The study found that parents of children with ADHD experience severe economic challenges and this is consistent with findings of previous studies. For instance, Fridman et al. [60] found that parents of children with ADHD are likely to quit their jobs to take care of their children. Kvist et al. [61] also concluded that having a child with ADHD will decrease the labor supply of parents. This is likely to put parents to severe economic strain. Studies conducted in Ethiopia [62, 63] found that parents of children with neurodevelopmental disorders experience such economic challenges as financial difficulty, lack of education and training, lack of financial support and employment opportunities.

Parents of children with ADHD also experience several psychological challenges. The current study revealed that what constantly worries parents is that who, in a highly stigmatized society, would have the patience to take care of their children in their absence. Cheung & Theule [64] and Durukan et al. [65] found a higher prevalence of depression and anxiety than parents of children without ADHD. This was similar to the experiences of some of the respondents in the present study. The present finding is in line with the study by Deault [66] that parents of children with ADHD have stress on which the children might play a role. In addition, Minichil et al. [67] found out that parents of children with mental health conditions can go through depression due to low social support which is also prevailing in this study. In another study, parents of children with ADHD reported that they experience greater levels of parenting stress than parents of children with autism [68] or with serious conditions such as Epilepsy [69]. Another study revealed that the distress is related to isolation, stigma, and frustration due to the lack of support [80].

Studies conducted in Ethiopia [65–67] found that the psychological challenges of parents of children with ASD were stress, concern about the child’s future, and psychological burden. According to Negash [59] uncertainty about the future, emotional disturbance, and spiritual crises were the challenges of parents who have children with intellectual disability. Tanzanian parents who have children with mental disorders including ADHD experience stress, sadness, bitterness and concern about the present as well as the future life of their children [53]. Three-fourths of parents in Nepal who have children with intellectual disability suffer from severe stress to clinically significant stress caused by their children’s disorder [70].

The present study adopted the Bronfenbrenner’s Ecological Model as its theoretical model. This theory describes the existence of multifaceted levels of the environment with an effect on the development of a child [29]. The theory demonstrates the development of a child within a system, the interaction between the systems, and the influences they have on each other and the child. According to the views of the theory, a complex system of relationships affected by multiple levels of the surrounding environment is where a child develops. When we compare the ecological model with all the challenges parents of children with ADHD are going through, here are some of our observations. At the microsystem level, it was found that the low level of knowledge the teachers have about ADHD has an impact on the parents. The reaction of some church members about the behaviour of a child with ADHD and the humiliation it has on the parents exhibit the bi-directional influence of the microsystems. At the exosystem, what was observed were the interactions of the parents with their neighbors, with friends of the parents, with their in-laws and relatives, and the way our mass media wrongly portrays mental health. At the macrosystem level, it was observed how these parents were affected by the stigma that exists about mental health. At the chronosystem level, the experience of some parents going through divorce, separation and an incident where a mother was forced to relocate from place to place due to her child’s condition was encountered. In addition, as explained by the ecological theory, the child will lack the means to explore other parts of the environment if the interaction in the immediate microsystem breaks down. The absence of acceptance in the child/parent (child/other significant adult) relations will make the children look for attention in an improper place. These inadequacies appear during adolescence as anti-social behaviour, absence of directing oneself and self-control [29]. This was somehow partially evident with the two parents who have teens with ADHD.

For the majority of the parents, adaptive strategies like religion, optimism, guidance from professionals and family support are used as coping mechanisms. Almost all of the parents use prayer as a coping strategy. Parents reported that they feel less stressed and believe that better days will come after praying. Healthcare providers’ guidance is also another coping mechanism that most parents use to deal with their challenges. The support from family members also played a role as a coping strategy. Research has revealed that religion is used to going through unpleasant experiences and trying to make meaning out of them and finally coming up with an optimistic outlook [71]. A Tanzanian study found that support from professionals, spiritual help from traditional healers and religious leaders, and assistance in child care from other family members were the coping mechanisms used by parents who have children with mental disorders including ADHD [53]. For parents of children with ASD, trying to make meaning out of the situation, support groups, being optimistic and religiosity were used as coping mechanisms [56]. Another similar study found that religion, social support, increased knowledge of autism, acceptance and cherishing little progress are the coping mechanisms for parents who have children with ASD [58].

Parents in the present study use optimism as a coping mechanism and this is consistent with the finding of a study by Oelofsen and Richardson [72] which revealed that parents of children with ADHD used an optimistic belief of having control over the situation, high sense of coherence and support as coping strategies. Parents from Hong Kong use acceptance, problem-centered coping methods and situational-based acts as their coping strategies [73]. A Nepalese study revealed that coping strategies used by Nepalese parents who have children with intellectual disability were acceptance, societal support, positive reinterpretation and growth, planning, inhibition of competing actions and use of emotional social support [70]. A similar study in Ethiopia found that spiritual beliefs, hope (better future) and relationships with other similar parents were the coping mechanisms of parents of children with intellectual disability [59].

Other parents use maladaptive coping strategies like social avoidance as a coping mechanism for the challenges they face. Avoidance coping strategies take place when stressful circumstances, experiences, or complicated opinions and feelings are averted to use as a coping strategy. This study is in line with one study that revealed that mothers who have children with ADHD use avoidance as one of their coping strategies [74].

### Strengths and limitations

We substantiated the views of parents who have children with ADHD by interviewing healthcare providers who are providing treatment to children with ADHD. We also used different methods of data collection (i.e. in-depth interviews and FGD) for triangulation purpose. Nevertheless, the findings of this study need to be interpreted taking several limitations into account. This is a qualitative study and generalizability of the findings of the study to other populations and study setting would not be possible. The participants were recruited from one government hospital and those who are attending private health centers might have different experiences. All respondents are from Addis Ababa and their experiences might be different from those living in the rural areas where the stigma is believed to be higher. In addition, the majority of the participants of the study are mothers and the experiences reported in the study may not represent fathers who have children with ADHD.

## Conclusions

Parents of children with ADHD experience various psychological, social and economic challenges. Support from healthcare professionals, family members and society at large plays a role for parents to cope with these challenges. Knowledge about ADHD prior to the diagnosis of the child has helped parents to easily accept the condition. Easy acceptance of the diagnosis reduces the psychological challenges of the parents. By creating awareness to society, most of the challenges of parents who have children with ADHD can be minimized.

The concerned government body has to consider the financial constraints parents are facing and facilitate further medication subsidies not only for children with ADHD but also for children with other neurodevelopmental disorders. This will encourage parents who intend to discontinue their children’s follow-up because of financial constraints to reconsider their intention. Healthcare facilities diagnosing and treating children with ADHD need to see the desperate need to facilitate for the parents to set up a support group where they would be able to exchange their experiences with like parents which in turn serve as a coping mechanism.

Policymakers need to observe the lack of teachers’ awareness about ADHD and the skill to handle children with ADHD as one of the challenges for parents of children with ADHD and work to design strategy to provide training that would equip teachers with the necessary knowledge in dealing with students with ADHD. As per the narratives of some of the parents, they are in constant brawls with their children who just became adolescents over their refusal to take medication. This can be an area for further study. Another focus for future research can be evaluating interventions that would help parents with ADHD cope with the challenges they experience. Quantitative studies that would estimate the burden and associated factors of parents who have children with ADHD are also warranted.

### Electronic supplementary material

Below is the link to the electronic supplementary material.

Supplementary Material 1

## Data Availability

The data used for this analysis will become available through the first author at any time from now up on reasonable request.

## Abbreviations

ADHD: Attention Deficit Hyperactivity Disorder
APA: American Psychological Association
ASD: Autism Spectrum Disorder
DSM: Diagnostic and Statistical Manual of Mental Disorders
FGD: Focus Group Discussion
SPHMMC: St. Paul Hospital Millennium Medical College
WHO: World Health Organization
