# Supplementary material for: Challenges and coping mechanisms of parents of children with attention deficit hyperactivity disorder in Addis Ababa, Ethiopia: a qualitative study
Source: BMC Psychol. 2024 Jun 17;12:354. doi: 10.1186/s40359-024-01828-0 (PMC11184847; doi:10.1186/s40359-024-01828-0)
Supplement: Supplementary file 1 — Supplementary Material 1 [file 40359_2024_1828_MOESM1_ESM.docx]

**Addis Ababa University**

**College of Education and Behavioral Studies**

**School of Psychology**

**1) Interview guide for Parents**

**Part I: Socio-demographic characteristics**

1. Is the parent being interviewed a biological or adopting parent?
2. Is the parent being interviewed father or mother?
3. Marital status: ______________________________
4. Age: _____________________________
5. Religion: _____________________________
6. Educational level**: _____________________________**
7. Occupation**: __________________________________**

**Part II: Questions on the lived experiences of parents**

1. How did you feel when you were first told about your child’s ADHD diagnosis?

- Denial
- Shame
- Anger
- Confusion
- Hopelessness
- Guilt
- Self-blame
- Acceptance

1. How does the ADHD diagnosis of your child change your life?
2. Improved your awareness about ADHD
3. Helped you to provide better care to your child
4. Helped you to change yourself to give better care to your child
5. What are your positive experiences while parenting a child with ADHD?

- Gained knowledge on the subject
- Opportunity to help other parents
- Advocate for children with ADHD
- Helped you not to be judgmental about children with other developmental conditions

1. What are the challenges you face in your daily life while parenting a child with ADHD?

- Stress
- Unavailability of reading material on ADHD
- Managing the child’s behavior on daily basis
- School work like home work, studying for exam and projects
- Being concerned about the future of the child
- Stigma
- Less social support
- Strained relationship with others
- Teachers lack of knowledge on ADHD
- Exhaustion
- Conflict with siblings
- Impact on your job
- Marital conflict
- Depression

1. What coping mechanisms do you use to deal with the challenges you face while parenting a child with ADHD?

- Seeking professional help
- Becoming optimistic
- Family support Religion (e.g. prayer)
- Social avoidance
- Experience sharing with likewise parents
- Acceptance and compassion
- Support from others

1. Is there anything that you would like to add?

**2) Focus group discussion (FGD) with parents**

**Part I: Socio-demographic characteristics**

1. Is the parent involved in the FGD a biological or adopting parent?
2. Is the parent involved in the FGD a father or mother?
3. Marital status: ______________________________
4. Age: _____________________________
5. Religion: _____________________________
6. Educational level**: _____________________________**
7. Occupation**: __________________________________**

**Part II: Questions on the lived experiences of parents**

1. How did you feel when you were first told about your child’s ADHD diagnosis?

- Denial
- Shame
- Anger
- Confusion
- Hopelessness
- Guilt
- Self-blame
- Acceptance

1. How does the ADHD diagnosis of your child change your life?

- Improved your awareness about ADHD
- Helped you to provide better care to your child
- Helped you to change yourself to give better care to your child

1. What are your positive experiences while parenting a child with ADHD?

- Gained knowledge on the subject
- Opportunity to help other parents
- Advocate for children with ADHD
- Helped you not to be judgmental about children with other developmental conditions

1. What are the challenges you face in your daily life while parenting a child with ADHD?

- Stress
- Unavailability of reading material on ADHD
- Managing the child’s behavior on daily basis
- School work like assignments, studying for exam, project
- Being concerned about the future of the child
- Stigma
- Less social support
- Strained relationship with others
- Teachers lack of knowledge on ADHD
- Exhaustion
- Conflict with siblings
- Impact on your job
- Marital conflict
- Depression

1. What coping mechanisms do you use to deal with the challenges you face while parenting a child with ADHD?

- Seeking professional help
- Becoming optimistic
- Family support Religion (e.g. prayer)
- Social avoidance
- Experience sharing with likewise parents
- Acceptance and compassion
- Support from others

1. Is there anything that you would like to add?

**3) Interview guide for healthcare professionals**

**Part I: Socio-demographic characteristics**

1. Sex: _________________________________
2. Age: _____________________________
3. Marital status: ______________________________
4. Religion: _____________________________
5. Educational level**: _____________________________**
6. Occupation**: __________________________________**

Part II: Questions on the experiences of parents who have children with ADHD

1. What do you observe in parents when they are first told about their child’s ADHD diagnosis?

- Denial
- Shame
- Anger
- Confusion
- Hopelessness
- Guilt
- Self-blame
- Acceptance

1. What are the challenges that parents face in parenting a child with ADHD?

- Stress
- Unavailability of reading material on ADHD
- Managing the child’s behavior on daily basis
- School work like assignment, studying for exams, project
- Being concerned about the future of the child
- Stigma
- Less social support
- Strained relationship with others
- Teachers lack of knowledge on ADHD
- Exhaustion
- Conflict with siblings
- Impact on your job
- Marital conflict
- Depression

1. As a health professional, how do you support or what kind of support you provide to parents who have children with ADHD diagnosis?

- Providing them with reading materials on ADHD
- Setting up support group
- Giving awareness sessions
- Facilitating a way they can get medication
- Providing psycho-education and counseling Recommending or referring school for the child
